# OP9 Bone Marrow Stroma Cells Differentiate into Megakaryocytes and Platelets

**DOI:** 10.1371/journal.pone.0058123

**Published:** 2013-03-01

**Authors:** Yumiko Matsubara, Yukako Ono, Hidenori Suzuki, Fumio Arai, Toshio Suda, Mitsuru Murata, Yasuo Ikeda

**Affiliations:** 1 Department of Laboratory Medicine, Keio University School of Medicine, Tokyo, Japan; 2 Division of Hematology, Department of Medicine, Keio University School of Medicine, Tokyo, Japan; 3 Department of Morphological and Biomolecular Research, Nippon Medical School, Tokyo, Japan; 4 Department of Cell Differentiation, Keio University School of Medicine, Tokyo, Japan; 5 Faculty of Science and Engineering, Life Science and Medical Bioscience, Waseda University, Tokyo, Japan; Johns Hopkins Univ. School of Medicine, United States of America

## Abstract

Platelets are essential for hemostatic plug formation and thrombosis. The mechanisms of megakaryocyte (MK) differentiation and subsequent platelet production from stem cells remain only partially understood. The manufacture of megakaryocytes (MKs) and platelets from cell sources including hematopoietic stem cells and pluripotent stem cells have been highlighted for studying the platelet production mechanisms as well as for the development of new strategies for platelet transfusion. The mouse bone marrow stroma cell line OP9 has been widely used as feeder cells for the differentiation of stem cells into MK lineages. OP9 cells are reported to be pre-adipocytes. We previously reported that 3T3-L1 pre-adipocytes differentiated into MKs and platelets. In the present study, we examined whether OP9 cells differentiate into MKs and platelets using MK lineage induction (MKLI) medium previously established to generate MKs and platelets from hematopoietic stem cells, embryonic stem cells, and pre-adipocytes. OP9 cells cultured in MKLI medium had megakaryocytic features, i.e., positivity for surface markers CD41 and CD42b, polyploidy, and distinct morphology. The OP9-derived platelets had functional characteristics, providing the first evidence for the differentiation of OP9 cells into MKs and platelets. We then analyzed gene expressions of critical factors that regulate megakaryopoiesis and thrombopoiesis. The gene expressions of p45NF-E2, FOG, Fli1, GATA2, RUNX1, thrombopoietin, and c-mpl were observed during the MK differentiation. Among the observed transcription factors of MK lineages, p45NF-E2 expression was increased during differentiation. We further studied MK and platelet generation using p45NF-E2-overexpressing OP9 cells. OP9 cells transfected with p45NF-E2 had enhanced production of MKs and platelets. Our findings revealed that OP9 cells differentiated into MKs and platelets *in vitro*. OP9 cells have critical factors for megakaryopoiesis and thrombopoiesis, which might be involved in a mechanism of this differentiation. p45NF-E2 might also play important roles in the differentiation of OP9 cells into MK lineages cells.

## Introduction

Platelets play critical roles in hemostatic plug formation and thrombosis [1]–[3]. Platelets are released from terminally differentiated megakaryocytes (MKs). The underlying molecular mechanisms of megakaryopoiesis and subsequent thrombopoiesis are only partially understood [4]–[10]. Current advances in a culture system to generate MKs and platelets *in vitro* help us to clarify the mechanism underlying MK differentiation and platelet production [11]. Also, studies on new strategies to manufacture MKs and platelets pursue to develop a donor-independent source for platelet transfusion [11].

MKs and platelets have been differentiated from hematopoietic stem cells (HSCs), embryonic stem (ES) cells, fetal liver cells, induced pluripotent stem (iPS) cells, and fibroblasts transfected with a combination of p45NF-E2, Maf G, and Maf K, *in vitro* [12]–[19]. Moreover, we reported the generation of MKs and functional platelets from both normal human subcutaneous adipose tissues and mouse pre-adipocyte cell line 3T3-L1 [20]–[22]. Differentiation of pre-adipocytes into MKs and platelets has been observed when the culture medium is switched from maintenance medium to MK lineage induction (MKLI) medium previously established to generate platelets from HSCs and ES cells [21]–[24].

The mouse bone marrow stroma cell line OP9 was established from macrophage colony stimulating factor deficient osteopetrotic mice [25]. OP9 cells have been widely used as feeder cells for the differentiation of ES cells and iPS cells into hematopoietic cells as well as for the differentiation of these stem cells into MKs and platelets [12]–[15], [25]–[27]. Also, OP9 cells were reported to be pre-adipocytes [28], [29]. In the present study, we examined whether OP9 cells differentiate MKs and platelets, based on our previous observations that pre-adipocytes differentiated into MKs and platelets [20]–[22], and then investigated the involved mechanisms of MK differentiation and platelet production from OP9 cells.

## Methods

### Cell Culture for Differentiation into MKs and Platelets

OP9 cells were maintained as described previously [12]. To induce the differentiation of OP9 cells into MK lineages, OP9 cells were cultured for 12 to 14 days using MKLI medium comprised of Iscove’s Modified Dulbecco’s Medium supplemented with 2 mM L-glutamine, 100 U/ml penicillin G sodium, 0.1 mg/ml streptomycin sulfate, 0.5% bovine serum albumin, 4 µg/ml LDL cholesterol, 200 µg/ml iron-saturated transferrin, 10 µg/ml insulin, 50 µM 2-β-mercaptoethanol, 20 µM each nucleotide (ATP, UTP, GTP, and CTP), and 50 ng/ml thrombopoietin (TPO; a gift from Kyowa Hakko Kirin CO., Ltd.) [19]–[24]. Mouse ES cells (gifted by Dr. Niwa H, RIKEN Center, Kobe, Japan) were established as described previously [30]. We maintained the ES cells as described previously [21], [31]. Primary mouse low-density bone marrow mononuclear cells (mBMMNCs) were obtained as described previously [23] and were then cultured in MKLI medium.

### Flow Cytometric Analyses

Surface marker analyses were performed on OP9 cells before the MK induction (day 0) and OP9 cells cultured in MKLI medium (OP9-derived cells). We used the directly labeled fluorescein isothiocyanate (FITC)-conjugated antibodies for CD41 (also known as platelet glycoprotein IIb) (BD bioscience), and CD42b (also known as platelet glycoprotein Ib alpha) (EMFRET Analytics Gmbh and Co.). The positive values (%) were calculated using cell number of binding to isotype control and cell number of binding to anti-CD41-antibody or anti-CD42b-antibody. Fluorescence-activated cell sorting was used to obtain CD41+ population from OP9-derived cells. OP9-derived MKs and platelets were counted as large-sized CD41+ cells and small-sized CD41+ cells, respectively [23]. The “platelet-sized” particles were defined using mouse platelets, and the plot is shown in Figure S1. DNA ploidy was assessed by interaction with propidium iodide (Sigma) as described previously [22].

### Morphologic Analyses

The ultrastructure for OP9-derived CD41+ cells on day 8 and mouse bone marrow cells was analyzed. These studies were done by transmission electron microscopy as described previously [32]. To examine proplatelet formation in OP9-derived cells, the OP9-derived CD41+ cells were plated on the fibrinogen-coated glass (100 µg/ml) were incubated for 6 hours at 37 degree C. These cells were observed using scanning electron microscopy as described previously [33]. Also, the cells were fixed with 4% paraformaldehide in Ca^++^- and Mg^++^-free Phosphate Buffered Saline (PBS) for 15 minutes at room temperature and then permeabilized by 0.2% Triton X 100 in PBS for 5 minutes at room temperature. The samples for proplatelet formation were stained with phycoerythrin-conjugated antibody for CD41 (BD bioscience), unlabeled anti-alpha-tubulin antibody (Lab Vision Co.) and FITC-conjugated anti-rabbit antibody, and DAPI blue.

### Immunohistochemical Studies

To analyze the expression of von Willebrand factor (VWF) and P-selectin in OP9-derived CD41+ cells on day 8, the cells stimulated with 10 µM ADP and 10 µM epinephrine were plated on the fibrinogen-coated glass (100 µg/ml, coating concentration). The cells were fixed with 4% paraformaldehide in Ca^++^- and Mg^++^-free PBS for 10 minutes at room temperature and then permeabilized by 0.2% Triton X 100 in PBS for 5 minutes at room temperature. The samples from OP9-derived CD41+ cells were stained with FITC-conjugated anti-VWF antibody (EMFRET Analytics Gmbh and Co.) and FITC-conjugated anti-P-selectin antibody (EMFRET Analytics Gmbh and Co.) for 60 min at room temperature. Cells were also stained DAPI blue and Texas Red Phalloidin (Invitrogen).

### Functional Analyses for OP9-derived Platelets

Function for OP9-derived platelets was examined. The analyses of fibrinogen binding and P-selectin surface exposure after stimulation were performed on OP9-derived cells on day 12, as described previously [22]. FITC-conjugated anti-P-selectin antibody (EMFRET Analytics Gmbh and Co.) was used for the analysis of P-selectin surface exposure in the presence or absence of stimulation.

### Gene Expression Analyses

To carry out gene expression analyses by reverse transcription-polymerase chain reaction (RT-PCR), total RNA samples were prepared from OP9-derived cells on days 0, 4, and 8, mES cells, and mBMMNC-derived cells on days 0 and 4 after treatment with Trizol reagent (Invitrogen). cDNA samples with genomic DNA removal were obtained by QuaniTect Reverse Transcription (QIAGEN) or by QuaniTect Whole Transcriptome (QIAGEN) with DNase (Promega), according to manufacturer’s protocols. Primers of RT-PCR for GATA1, GATA2, Fli1, FOG, p45NF-E2, and GAPDH were used as described previously [16]. The pre-made primers (Applied Biosystems) were used for RT-PCR for OCT3/4, SOX2, RUNX1, TPO, c-mpl, KLF1, and PU1. Quantitative real time-PCR was also performed on samples from OP9 cells and OP9-derived cells using pre-made primers (Applied Biosystems). The amount of target normalized to GAPDH was determined by evaluating expression: 2^−ΔΔCt^, where ΔΔCt = ΔCts -ΔCt cb, ΔCt was threshold cycle, ΔCts was the ΔCt value of a sample, ΔCt cb was that of the calibrator and meant the difference in threshold cycles between the target and reference.

### Retroviral Vectors and Cell Culture

Retroviral vector for overexpression of p45NF-E2 in OP9 cells was used as described previously [19]. A CalPhos Mammalian Transfection Kit (Clontech) and AmphoPack-293 cells (Clontech), as packaging cells, were used according to the manufacture’s protocol. AmphoPack-293 cells were transfected with p45NF-E2 expression vector or empty vector. After 48 hours of transfection, retroviral supernatants were collected. OP9 cells were infected with the p45NF-E2 expression vector or empty vector (p45NF-E2-OP9 and empty-OP9). Each of cells was cultured in MKLI medium for 12 days.

### Statistics

Mean values of two groups were compared using Student’s t-test. Statistical analysis was performed using StatView (ver 5.0, for Macintosh, SAS Institute Inc., Cary, NC). A p value of less than 0.05 was considered statistically significant.

## Results

### Differentiation of OP9 Cells into MKs and Platelets

We examined whether OP9 cells differentiate into MKs and platelets by using MKLI medium previously established to generate MKs and platelets from HSCs, ES cells, and pre-adipocytes. OP9 cells cultured for 6 days in MKLI medium resulted in adherent cells and a few floating cells. The OP9-derived cells on day 8 had adherent, loosely adherent, and floating cells, and most of cells on day 12 had floating cells (Figure S2A). Among OP9-derived cells on day 8, floating and loosely adherent cells morphologically resembling MKs derived from mBMMNCs cultured in MKLI medium (Figure S2B). These OP9-derived cells were characterized using surface markers, DNA polyploidy with nuclear staining, morphology using electron microscopy, and immunohistochemistry.

**Figure 1:**
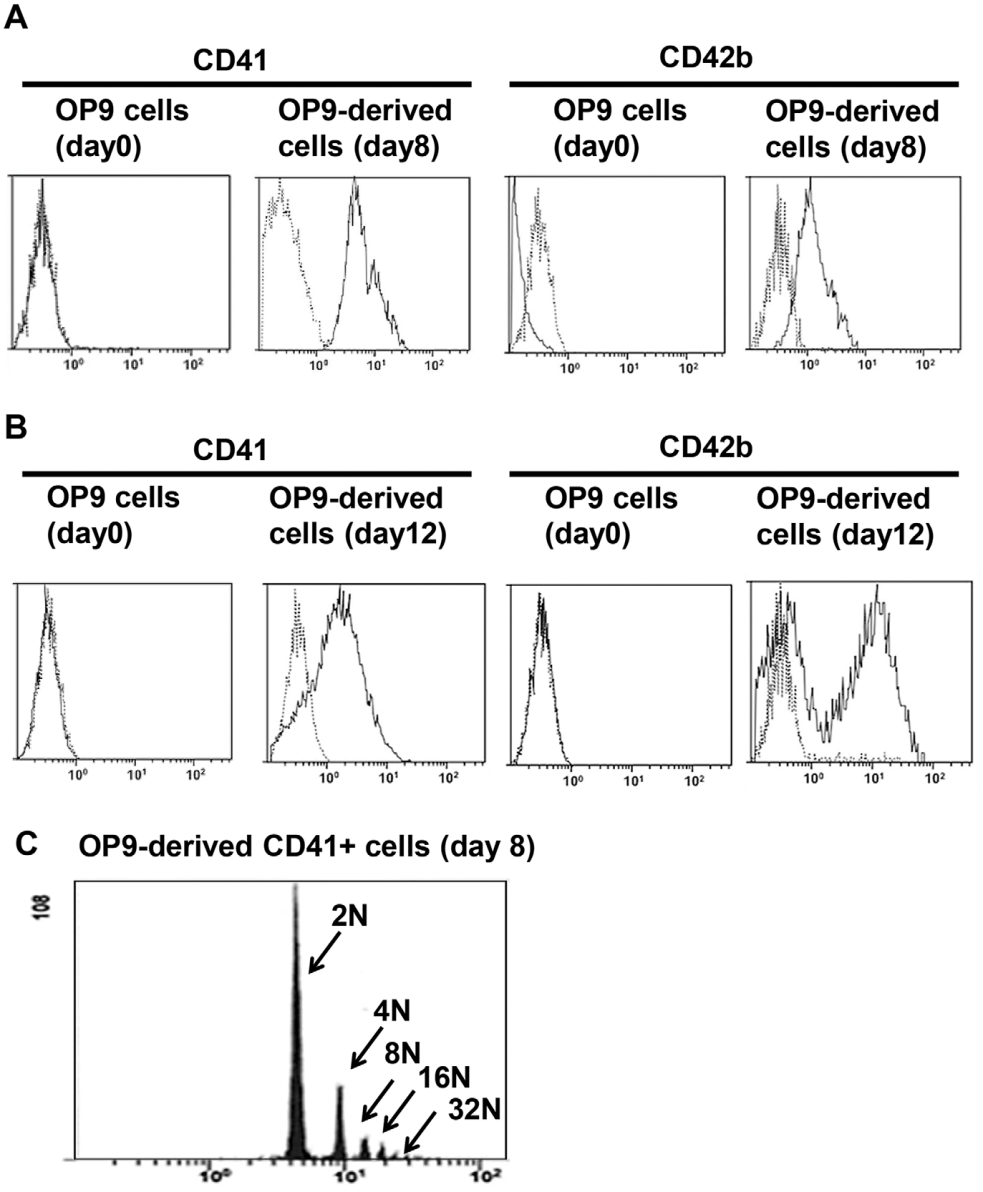
Flow cytometric analyses for megakaryocytes and platelets differentiated from OP9 cells. **A,** Representative flowcytometry histogram of CD41 and CD42b expression on megakaryocyte-sized cells. Dot-line shows data using isotype control. **B,** Representative flowcytometry histogram of CD41 and CD42b expression on platelet-sized cells. Dot-line shows data using isotype control. **C,** DNA ploidy analysis in OP9-derived CD41+ cells on day 8.

**Figure 2:**
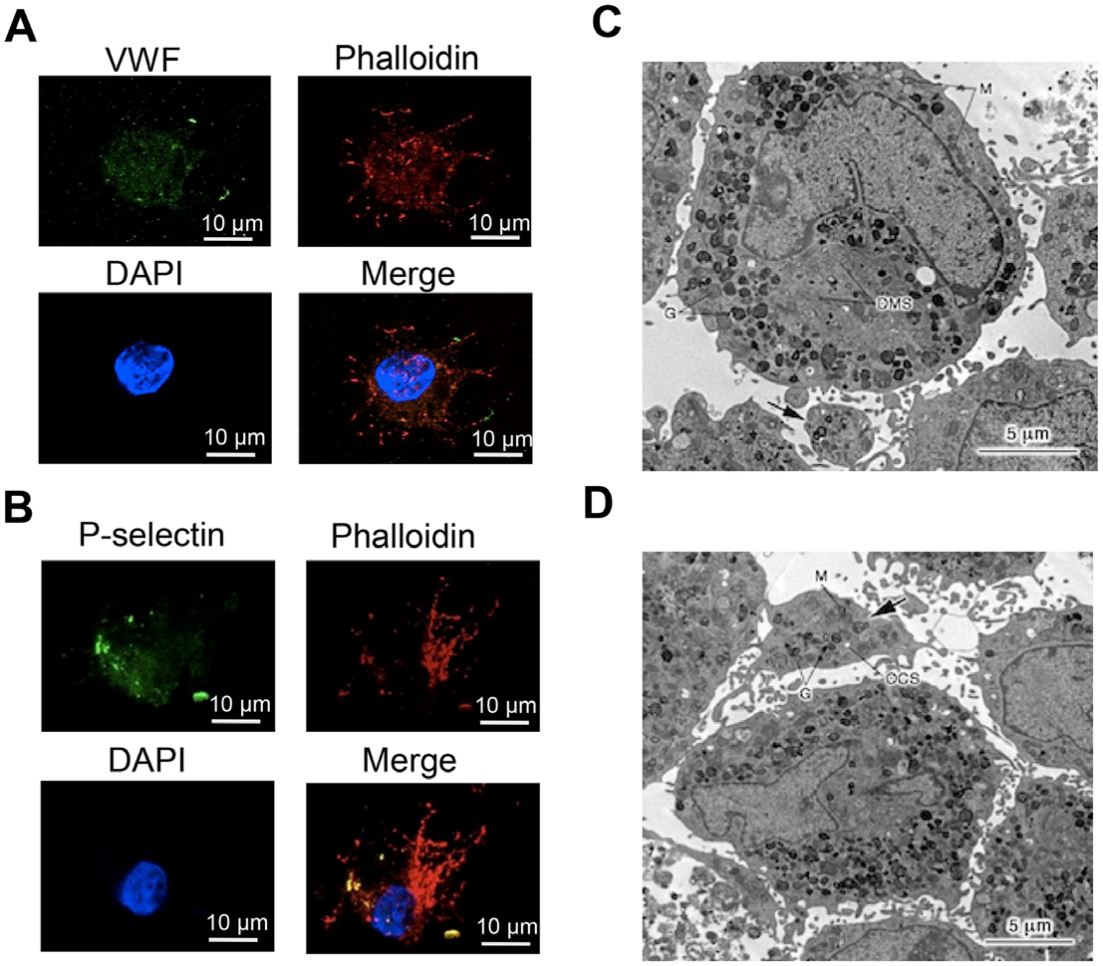
Cell staining and morphologic analyses. **A,** Cells stained with antibody for von Willebrand factor (green), Phalloidin (red) and DAPI (blue). **B**, Cells stained with antibody for P-selectin (green), Phalloidin (red) and DAPI (blue). **C,** **D,** Transmission electron micrograph of OP9-derived CD41+ cells. Megakaryocyte and platelet (arrow indicated in black): G, granule, DMS, demarcation membrane system, M, mitochondria, and OCS, open canalicular system.

**Figure 3:**
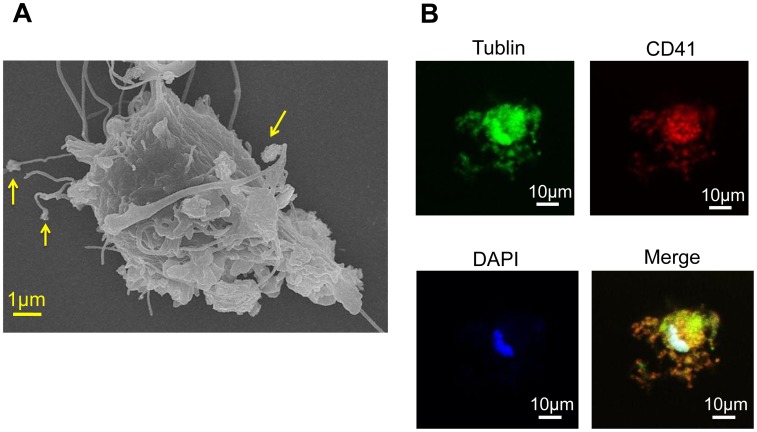
Proplatelet formation in OP9-derived megakaryocyte. **A.** Scanning electron micrograph of proplatelets (arrow indicated in yellow) forming OP9-derived CD41+ cells. **B.** Proplatelet-forming megakaryocyte was stained with antibody for CD41 (red) and alpha-tubulin (green), and DAPI (blue).

**Figure 4:**
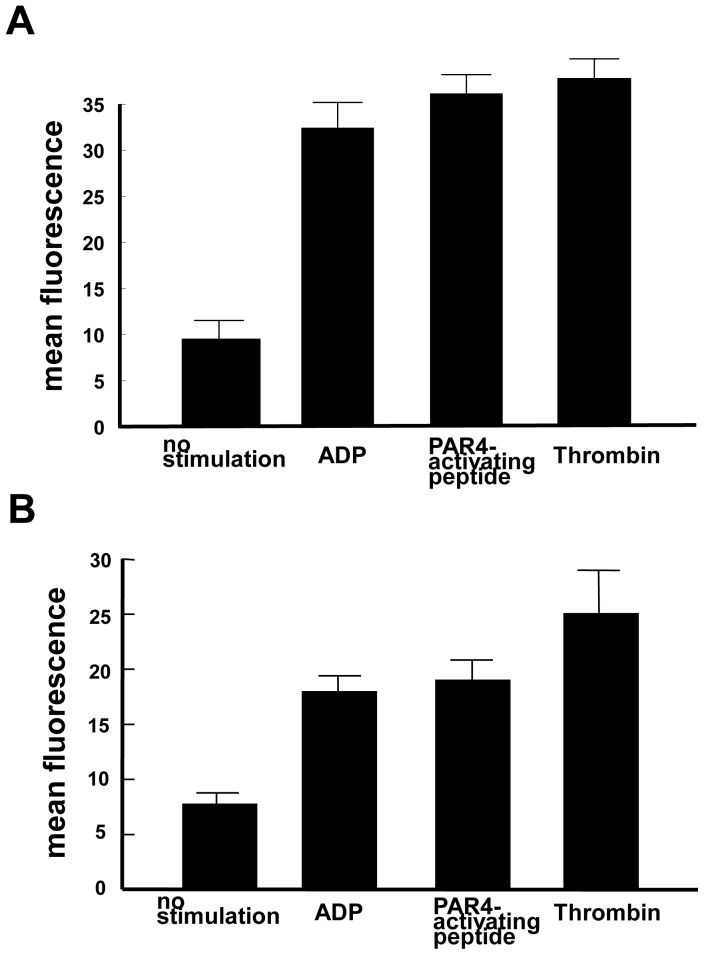
Functional analyses for OP9-derived platelets. **A,** By flowcytometry analysis, Alexa Fluor 488-labeled fibrinogen (100 µg/ml) binding to platelets derived from OP9 cells was examined in the presence or absence of platelet stimulation reagents. **B,** The surface exposure of P-selectin was analyzed in the presence or absence of platelet stimulation reagents.

By flow cytometric analysis with MK lineage specific markers, approximately 95% and 60% of these OP9-derived MK-sized cells on day 8 expressed CD41, a surface marker throughout MK differentiation, and CD42b, a surface marker for the late stage of MK differentiation, respectively (Figure 1A). Approximately 70% and 60% of OP9-derived platelet-sized cells on day 12 expressed CD41 and CD42b, respectively (Figure 1B). Regarding the number of the OP9-derived MKs and platelets, approximately 4×10^4^ MKs and 1×10^5^ platelets were generated from 1×10^6^ OP9 cells before the MK induction. OP9 cells before the MK induction (day 0) did not express CD41 and CD42b (Figure 1A, 1B). DNA ploidy of OP9-derived CD41+ cells ranged from 2N to 32N (Figure 1C). Immunohistochemical analyses showed that VWF and P-selectin, cytoplasmic proteins of MK lineage cells, were positive in OP9-derived CD41+ cells (Figure 2A, 2B). Under electron microscopic observation, OP9-derived MK-sized CD41+ cells had typical organelles for MKs, such as granules, demarcation membrane system, and lobulated nuclei, and OP9-derived platelet-sized CD41+ cells showed typical features for platelets, such as granules, mitochondria, and open canalicular system (Figure 2C, 2D). The present observations were similar to what described in MKs derived from mBMMNCs (Figure S3). Also, the present observations were similar to what described in MKs and platelets derived from mouse ES cells, mouse pre-adipocyte cell line 3T3-L1, human bone marrow CD34-positive cells, and human adipose tissues [19], [20]. OP9-derived MK-sized CD41+ cells were examined for proplatelet formation under scanning electron microscopic observation. We observed proplatelets forming OP9-derived MK-sized CD41+ cells (Figure 3A). Moreover, proplatelet-forming OP9-derived MK showing CD41 and alpha-tubulin was observed (Figure 3B). To examine whether OP9-derived platelets are functional, fibrinogen-binding assay was performed on OP9-derived platelet-sized cells on day 12. Binding of Alexa Fluor 488-labeled fibrinogen to OP9-derived platelet-sized cells was increased upon stimulation when assessed by mean fluorescence (mean±S.D.): 9.7±0.9 (no stimulation), 25.7±0.3 (10 µM ADP), p<0.0001 (vs no stimulation), 28.6±0.4 (1.5 mM PAR4-activating peptide), p<0.0001 (vs no stimulation), and 29.2±1.6 (0.5 U/ml thrombin), p<0.0001 (vs no stimulation) (Figure 4A). The representative data of dot-plots in this assay are shown in Figure S4. Moreover, P-selectin surface exposure, a marker for platelet activation, on OP9-derived platelet-sized cells on day 12 was examined in the presence or absence of stimulation, and mean fluorescence (mean±S.D.) was 7.8±1.1 (no stimulation), 17.9±1.5 (10 µM ADP), p = 0.0159 (vs no stimulation), 19.1±1.8 (1.5 mM PAR4-activating peptide), p = 0.0162 (vs no stimulation), and 25.1±3.8 (0.5 U/ml thrombin), p = 0.0162 (vs no stimulation) (Figure 4B). These observations indicated that OP9 cells differentiated into MKs and platelets *in vitro*.

### Analyses of Gene Expressions of OP9 Cells during MK Differentiation

**Figure 5:**
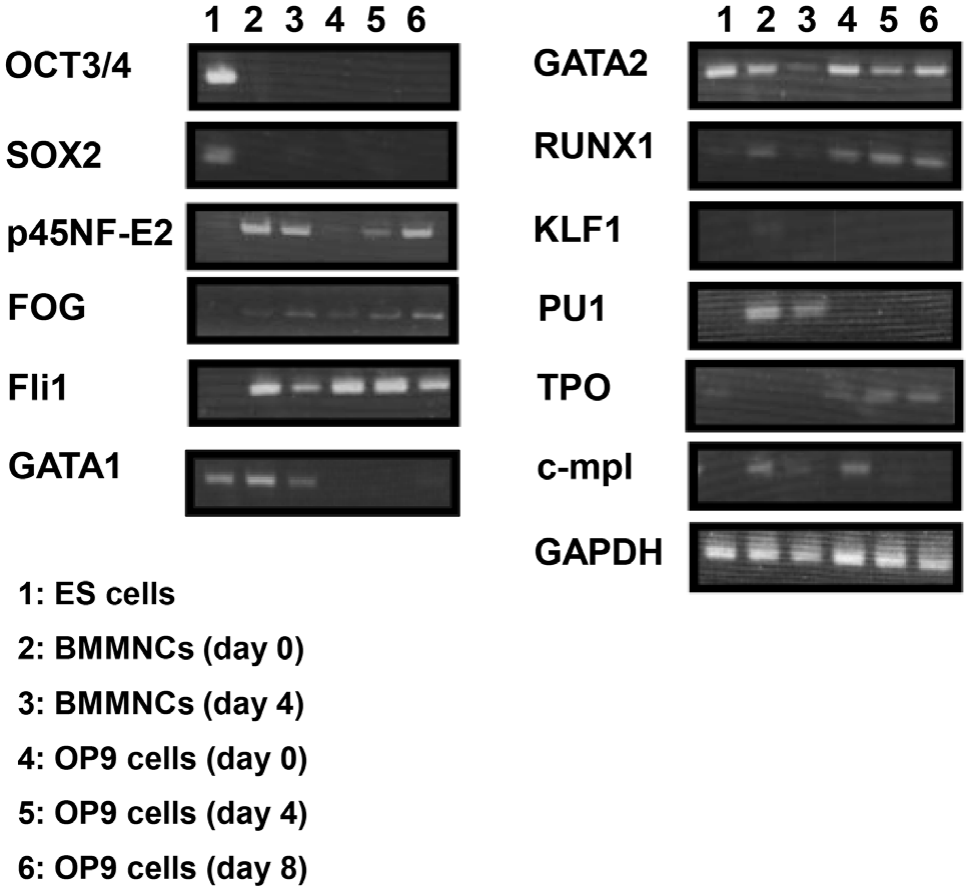
Analyses of gene expression of OP9 cells during megakaryocyte differentiation. Gene expression assessed by reverse transcription-PCR.

We then analyzed gene expression of candidate factors to elucidate mechanisms for the differentiation of OP9 cells into MKs and platelets. To examine whether this conversion goes through the status of cell pluripotency, the expressions of OCT3/4, specifically expressed in pluripotent cells, and SOX2, a key factor for the maintenance of cell pluripotency [34], [35], were analyzed. Expression of both OCT3/4 and SOX2 was not detected in OP9 cells (day 0) and OP9-derived cells by RT-PCR (Figure 5). We also analyzed the gene expressions for transcription factors, p45NF-E2, FOG, Fli1, GATA1, GATA2, and RUNX1, that regulate megakaryopoiesis and thrombopoiesis [5]–[11]. These transcription factors, except for GATA1, were clearly detected in OP9 (day 0) and OP9-derived cells (days 4 and 8) (Figure 5). Because major cell population of BMMNCs on day 4 morphologically resembled that of OP9-derived cells on day 8, we used BMMNCs on days 0 and 4 as a control. OP9 cells (day 0) also showed gene expression of TPO and c-mpl, receptor for TPO (Figure 5). Regarding the key transcription factors for other hematopoietic cell lineages, PU.1 for leukocytes [36] and KLF1 for erythrocytes [37], were not detected in OP9 cells (day 0) and OP9-derived cells (days 4 and 8) (Figure 5). Among the observed transcription factors, p45NF-E2 expression was increased during the differentiation of OP9 cells into MKs, and expression levels of p45NF-E2 in OP9 cells (day 0) and OP9-derived cells (day 4) were measured by quantitative real-time PCR analysis. It was found that expression levels of OP9-derived cells (day 4) had 3.91±0.08-fold higher than that of OP9 cells (day 0). Other transcription factors were also measured. It was for 1.54±0.02-fold higher for FOG, 0.03±0.00 (0.0007)-fold higher for Fli1, 0.20±0.03-fold higher for GATA2, and 1.00±0.16-fold higher for RUNX1, as compared with those of OP9 cells (day 0). TPO expression levels of OP9-derived cells (day 4) had 3.59±0.70-fold higher than that of OP9 cells (day 0). c-mpl expression levels of OP9-derived cells (day 4) had 0.182±0.11-fold higher than that of OP9 cells (day 0). These results indicate that OP9 cells possess critical factors for MK differentiation and platelet production. Among them, the p45NF-E2 expression was increased during the differentiation of OP9 cells into MK lineages.

### Enhanced Production of MKs and Platelets from OP9 cells Transfected with p45NF-E2

**Figure 6:**
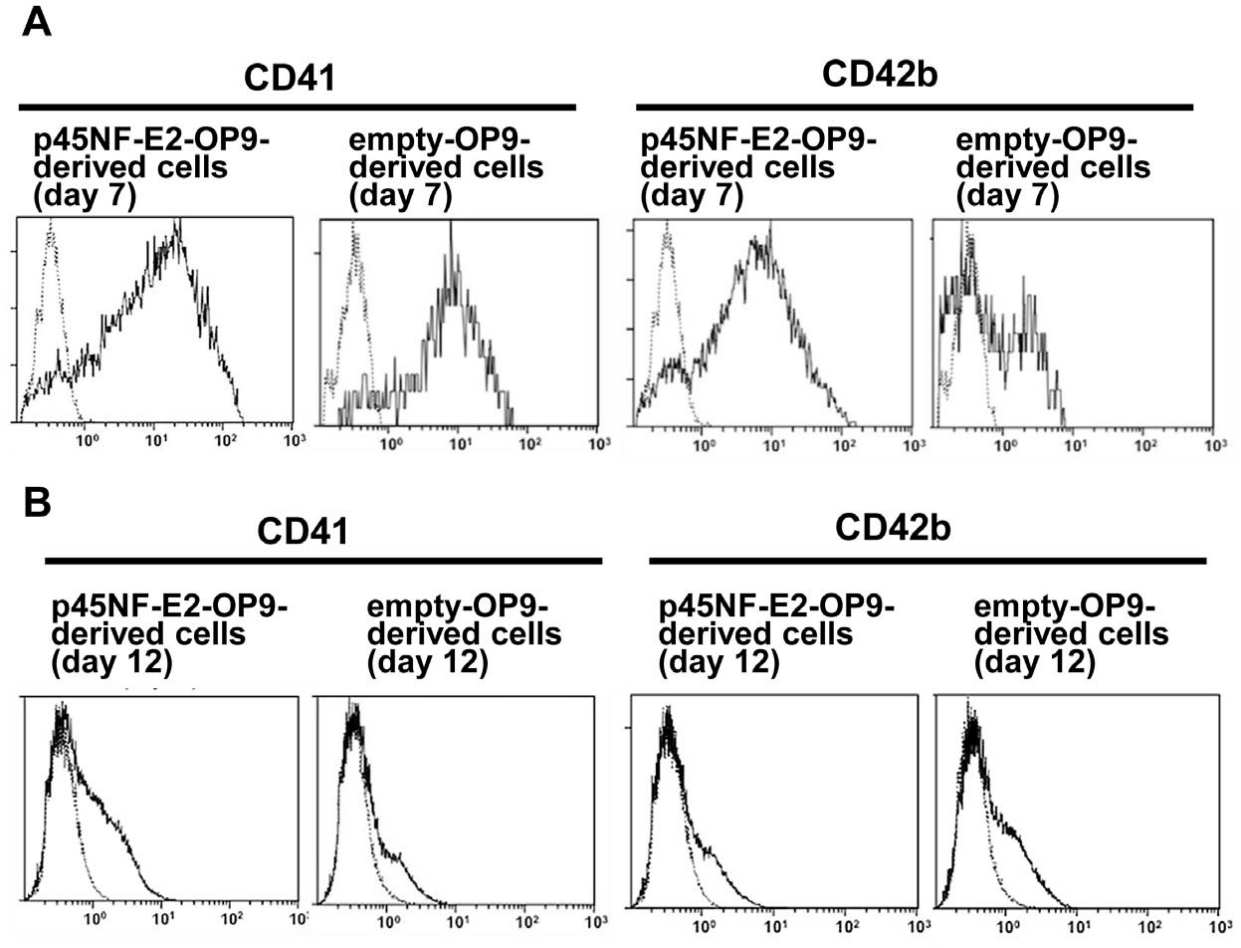
Production of megakaryocytes and platelets from OP9 cells transfected with p45NF-E2. **A,** Representative flowcytometry histogram of CD41 and CD42b expression on megakaryocyte-sized cells in each of samples introduced with the indicated vectors. Dot-line shows data using isotype control. **B,** Representative flowcytometry histogram of CD41 and CD42b expression on platelet-sized cells in each of samples introduced with the indicated vectors. Dot-line shows data using isotype control.

Based on increased expression of p45NF-E2 during differentiation of OP9 cells into MK lineages, we further studied the effect of OP9 cells transfected with p45NF-E2 on MK and platelet production. Transfection efficiency was estimated by DsRed expression, and approximately 50% of transfected OP9 cells was positive for DsRed expression, as assessed by flowcytometry (data not shown). Gene expression levels, assessed by qRT-PCR, of p45NF-E2 in p45NF-E2-OP9 cells on day 4 had 37.68±11.36-fold higher than that in empty-vector-OP9 cells. The p45NF-E2-OP9 cells and empty-vector-OP9 cells were cultured in MKLI medium for 12 days. By flow cytometric analysis, nearly all of the p45NF-E2-OP9-derived MK-sized cells on day 7 expressed CD41, whereas approximately 60% of empty-vector-OP9-derived MK-sized cells on day 7 expressed CD41 (Figure 6A). Also, CD42b expression was approximately 50% and 20% of p45NF-E2-OP9- and empty-vector-OP9-derived MK-sized cells on day 7, respectively (Figure 6A). The 1×10^6^ OP9 cells (day 0) generated 3.3±1.8×10^4^ MKs for p45NF-E2-OP9 cells and 2.2±1.6×10^4^ (p = 0.4008) MKs for empty-vector-OP9 cells. The effect of p45NF-E2-overexpressing OP9 cells on platelet production was also examined. CD41 expression was approximately 50% and 20% of p45NF-E2-OP9- and empty-vector-OP9-derived platelet-sized cells on day 12, respectively (Figure 6B), and CD42b expression was approximately 20% and 10% of p45NF-E2-OP9- and empty-vector-OP9-derived platelet-sized cells on day 12, respectively (Figure 6B). It was found that the number of p45NF-E2-OP9-derived platelet-sized CD41+ cells on day 12 showed approximately 3-fold higher than those in the empty-vector-OP9-derived platelet-sized CD41+ cells on day 12. The number of platelets generated from 1×10^6^ OP9 cells (day 0) was 2.9±2.6×10^5^ for the p45NF-E2-OP9-derived platelets and 1.0±7.3×10^5^ (p = 0.0518) for the empty-vector-OP9-derived platelets. These findings indicate that p45NF-E2 has a critical role in the generation of MKs and platelets from OP9 cells, although the production of MKs and platelets was not significantly different between the p45NF-E2-OP9 cells and empty-vector-OP9 cells.

## Discussion

This study demonstrated that OP9 cells differentiated into MKs and platelets *in vitro* using MKLI medium previously established to differentiate HSC, ES cells, pre-adipocytes into MK lineages. The present findings provide the first evidence for the differentiation of OP9 cells into MK lineages. Regarding the efficiency of the MK and platelet production from OP9 cells, approximately 4×10^4^ MKs and 1×10^5^ platelets were generated from 1×10^6^ OP9 cells. On the other hand, 1×10^6^ human bone marrow mononuclear cells produced approximately 6×10^3^ MKs and 3×10^3^ platelets in a similar culture scale using MKLI medium [24]. Although it is difficult to compare precisely the efficiency of the MK and platelet production among various stem cell sources, our observations suggested that OP9 cells possess high capacity of the differentiation into MK lineages *in vitro*.

OP9 cells cultured in maintenance medium express specific surface marker for MSC and were reported to be pre-adipocytes [28], [29]. These cell lineage fate of mesenchymal cells is distinct from that of HSCs, and OP9 cells are widely used as feeder cells for differentiation of iPS cells and ES cells into hematopoietic cells and MK lineages [12]–[15], [25]–[27]. However, the present study shows that OP9 cells themselves are the source of MKs and platelets. OP9-derived MKs and platelets were characterized by specific surface markers, DNA polyploidy, morphology using electron microscopy, and immunohistochemistry. These analyses have been performed on *in vitro*-generated MKs and platelets beginning with cell sources including HSCs, ES cells, and iPS cells. When MKs and platelets derived from iPS cells or ES cells are harvested in the differentiation study using OP9 co-culture system, there is a possibility that some of MK lineage cells are derived from OP9 cells.

The gene expression analyses indicated that differentiation of OP9 cells into MK lineages did not share the common mechanism with pluripotent cells. OP9 cells possess the important factors related to megakaryopoiesis and thrombopoiesis, and these observations are compatible with our previous findings that 3T3-L1 pre-adipocytes possess GATA2, RUNX1, Fli1, FOG1, and p45NF-E2. The expression of GATA1 was not detected in OP9 cells and 3T3-L1 cells [19]. Furthermore, we did not observe the GATA1 expression during differentiation of OP9 cells into MKs. Although GATA1 was reported to be a critical factor for the erythroid and MK development, previous studies demonstrated that GATA2 coordinates MK differentiation in GATA1 deficient and mutant cells [38]. Also, the present study revealed that the differentiation of OP9 cells into MK lineages involves in a p45NF-E2-mediated mechanism. The NF-E2 transcriptional factor is a basic-leucine zipper hetero-dimer complex consisting of p45 subunit, known as tissue-restricted subunit, and the small Maf proteins, Maf K and Maf G, known as widely expressed in many cells [39]–[45]. Observations in p45NF-E2 deficient MKs suggested that p45NF-E2 is important in the MK terminal differentiation and platelet release [46], [47]. On the other hand, the *in vitro* and *in vivo* study using p45NF-E2-overexpressing bone marrow cells showed additional roles of p45NF-E2 in early megakaryopoiesis [48]. We previously reported that fibroblasts transfected with p45NF-E2, Maf G and Maf K differentiated into MKs and platelets, whereas fibroblast did not differentiate into MK lineage cells. These observations support p45NE-E2, Maf G, and Maf K as critical factors for megakaryopoiesis and thrombopoiesis. In the present study, OP9 cells have Maf G and Maf K, and thus cells were transfected with P45NF-E2. The present findings provide additional information for the importance of p45NF-E2 in megakaryopoiesis and thrombopoiesis. Further studies are definitely needed to elucidate the detailed pathways that cause OP9 cells to differentiate into the MK lineage ultimately leading to platelet production.

In summary, OP9 cells differentiated into MKs and platelets, although OP9 cells have been wildly used as feeder cells in differentiation of ES cells and iPS cells into MKs and platelets. OP9 cells possess critical factors related to megakaryopoiesis and thrombopoiesis. The generation of MKs and platelets from OP9 cells could have important implications for study on the underlying mechanisms of megakaryopoiesis and thrombopoiesis.

## Supporting Information

Figure S1 **The plot of mouse platelets in flow cytometric analysis.**
(TIFF)

Figure S2 **Megakaryocyte lineage cells were generated from OP9 cells in vitro. A,** Schematic outline and pictures for OP9 cells and differentiated stages into megakaryocyte lineages. **B**, Mouse bone marrow mononuclear cells were cultured in megakaryocyte lineage induction media for 7 days.
(TIFF)

Figure S3 **Transmission electron micrograph of mouse bone marrow mononuclear cells.**
(TIFF)

Figure S4 **Alexa Fluor 488-labeled fibrinogen binding to platelets derived from OP9 cells was examined in the presence or absence of platelet stimulation reagents.**
(TIFF)
