# Supplementary figures and images for: OP9 Bone Marrow Stroma Cells Differentiate into Megakaryocytes and Platelets
Source: PLoS One. 2013 Mar 1;8(3):e58123. doi: 10.1371/journal.pone.0058123 (PMC3585802; doi:10.1371/journal.pone.0058123)

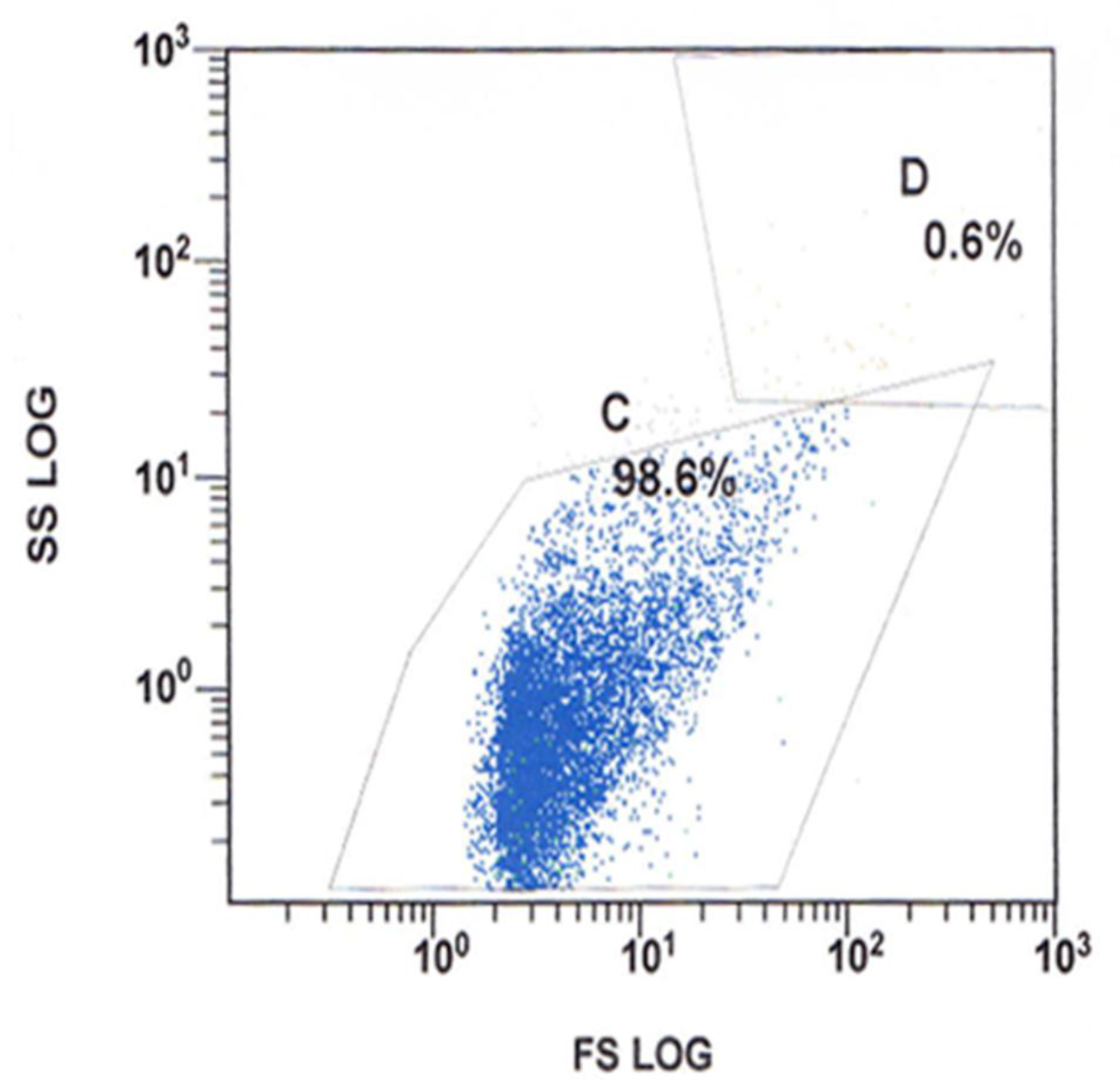

Supplement: Figure S1 — The plot of mouse platelets in flow cytometric analysis. (TIFF) [file pone.0058123.s001.tiff]

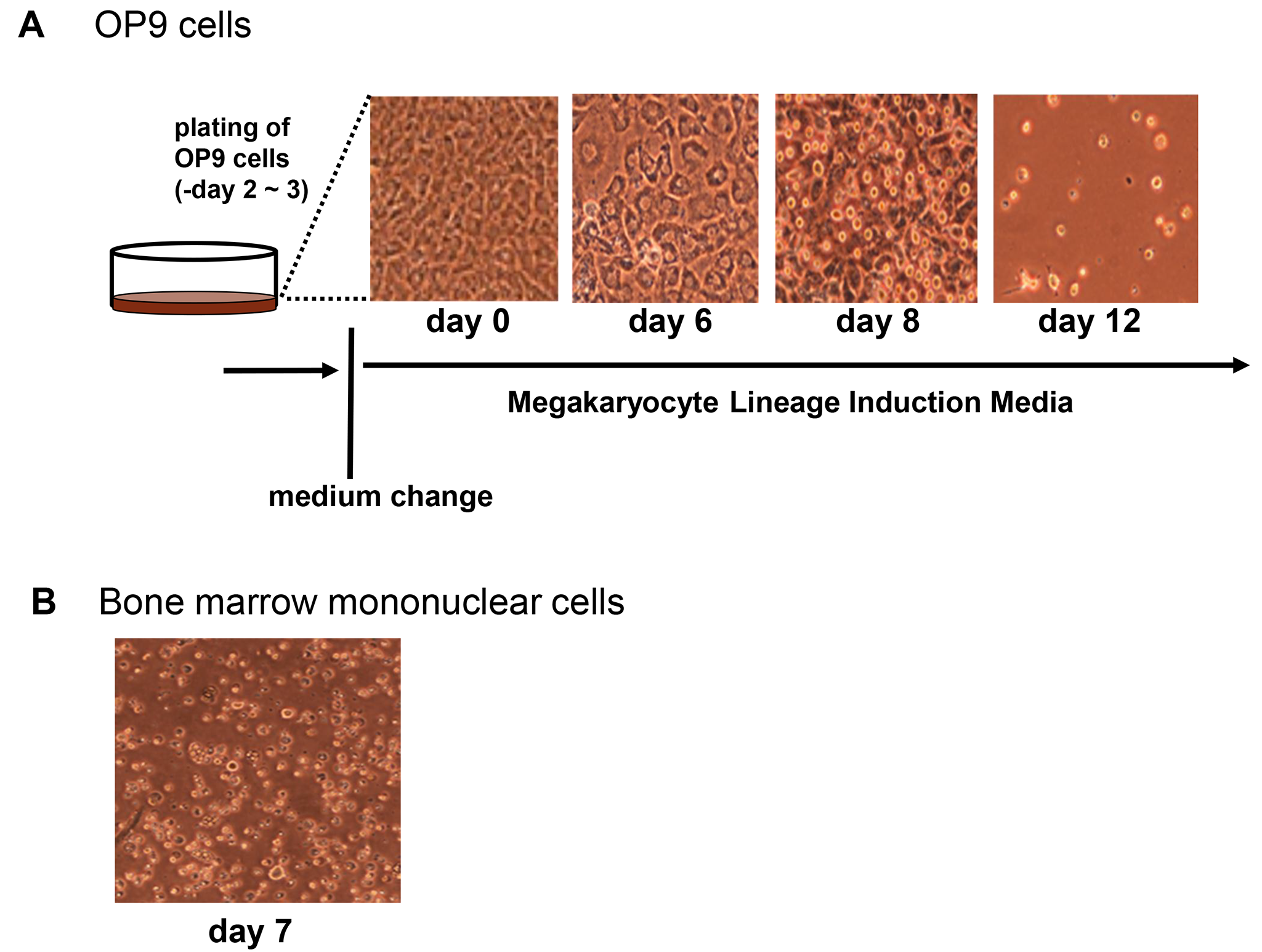

Supplement: Figure S2 — Megakaryocyte lineage cells were generated from OP9 cells in vitro. A, Schematic outline and pictures for OP9 cells and differentiated stages into megakaryocyte lineages. B, Mouse bone marrow mononuclear cells were cultured in megakaryocyte lineage induction media for 7 days. (TIFF) [file pone.0058123.s002.tiff]

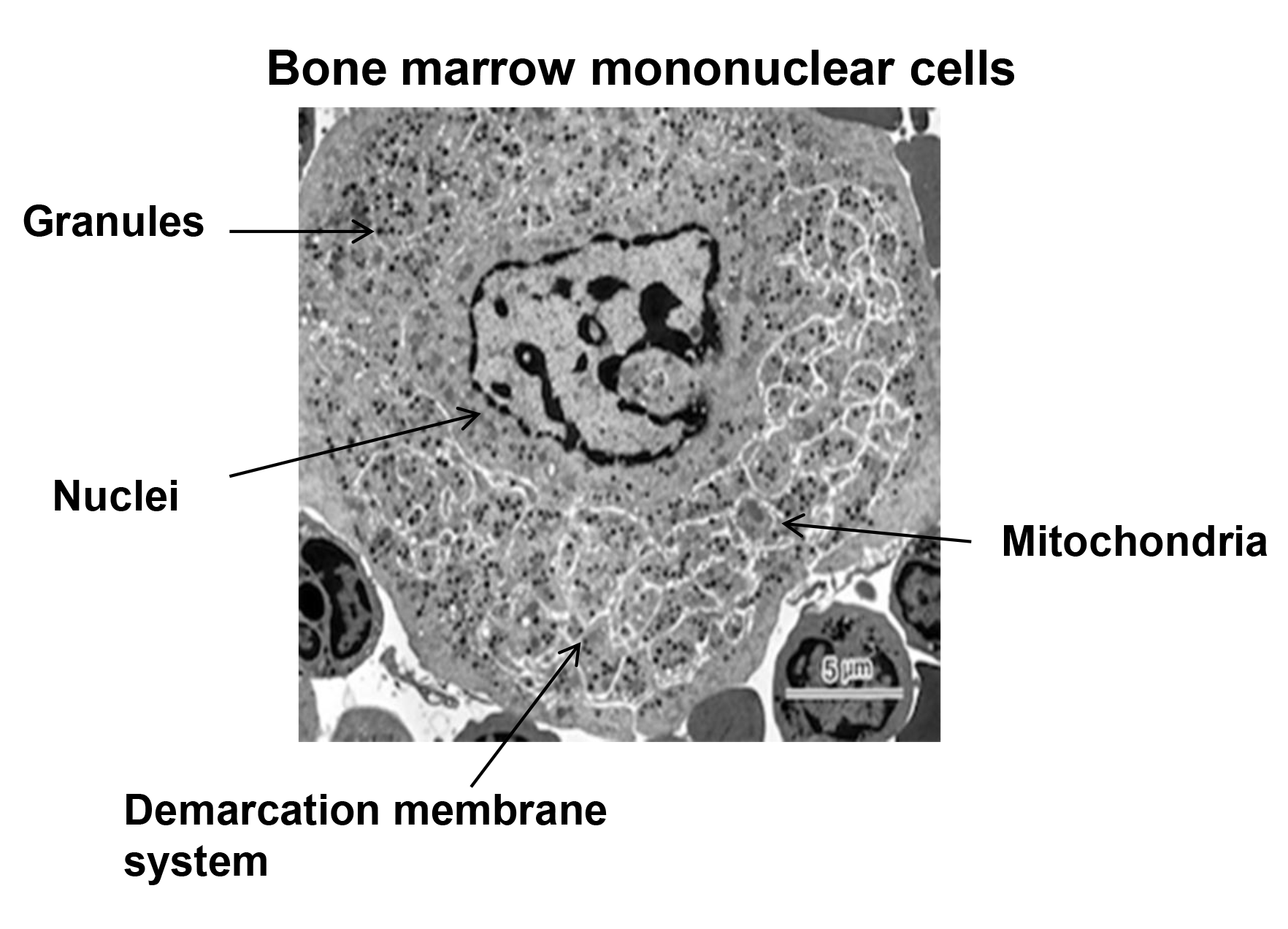

Supplement: Figure S3 — Transmission electron micrograph of mouse bone marrow mononuclear cells. (TIFF) [file pone.0058123.s003.tiff]

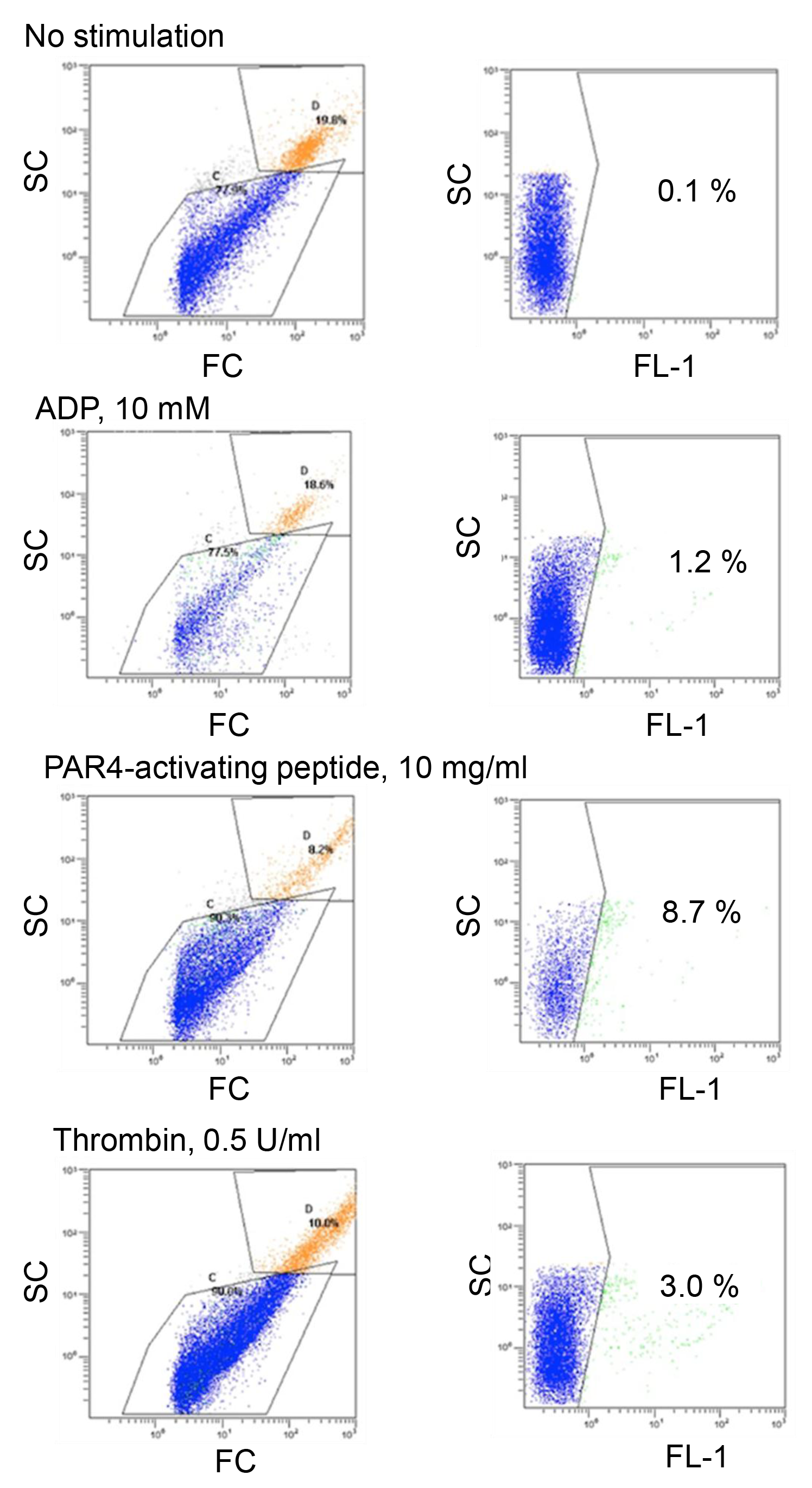

Supplement: Figure S4 — Alexa Fluor 488-labeled fibrinogen binding to platelets derived from OP9 cells was examined in the presence or absence of platelet stimulation reagents. (TIFF) [file pone.0058123.s004.tiff]
